# Contactless monitoring of respiratory rate variability in rats under anesthesia with a compact 24GHz microwave radar sensor

**DOI:** 10.3389/fvets.2025.1518140

**Published:** 2025-03-26

**Authors:** Guanghao Sun, Masaki Kurosawa, Yoshiki Ninomiya, Kohei Baba, Nguyen Huu Son, Hoang Thi Yen, Satoshi Suzuki, Yutaka Kano

**Affiliations:** ^1^Graduate School of Informatics and Engineering, The University of Electro-Communications, Chofu, Japan; ^2^Faculty of Radio-Electronic Engineering, Le Quy Don Technical University, Hanoi, Vietnam; ^3^Department of Mechanical Engineering, Kansai University, Osaka, Japan

**Keywords:** laboratory animal, microwave radar, anesthesia, respiratory rates, non-contact, non-invasive techniques, rat

## Abstract

**Objective:**

The objective of this study was to develop and validate a noncontact monitoring system for respiratory rate variability in rats under anesthesia using a 24GHz microwave radar sensor. This study aimed to address the need for stress-free monitoring techniques that comply with the 3Rs principle (Reduction, Replacement, and Refinement) in laboratory animal settings.

**Methods:**

Utilizing a 24GHz microwave radar sensor, this system detects subtle body surface displacements induced by respiratory movements in anesthetized rats. The setup includes a 24.05 to 24.25 GHz radar module coupled with a single-board computer, specifically Raspberry Pi, for signal acquisition and processing. The experiment involved four male Wistar rats tracking the variability in their respiratory rates at various isoflurane anesthesia depths to compare the radar system’s performance with reference measurements.

**Results:**

The radar system demonstrated high accuracy in respiratory rate monitoring, with a mean difference of 0.32 breaths per minute compared to laser references. The Pearson’s correlation coefficient was high (0.89, *p* < 0.05), indicating a strong linear relationship between the radar and reference measurements. The system also accurately reflected changes in respiratory rates corresponding to different isoflurane anesthesia levels. Variations in respiratory rates were effectively mapped across different anesthesia levels, confirming the reliability and precision of the system for real-time monitoring.

**Conclusion:**

The microwave radar-based monitoring system significantly enhanced the animal welfare and research methodology. This system minimizes animal stress and improves the integrity of physiological data in research settings by providing a non-invasive, accurate, and reliable means of monitoring respiratory rates.

## 1 Introduction

Laboratory animals, such as mice and rats, are used in life sciences research and contribute immeasurably to human health and welfare. Measurement of vital signs is important for the health management of laboratory animals based on the 3Rs (Reduction, Replacement, Refinement) principle (1, 2). Currently, the most commonly used devices for measuring respiration, heart rate, and core body temperature in laboratory animals are implantable sensors and contact-type devices that use electrodes (3, 4). However, methods that apply stress by contact are highly invasive to the animal body, and there is a risk that physiological information may be hidden owing to the influence of stress on the measurement results. Laboratory animal studies often involve surgical procedures or specific handling techniques that require anesthesia. Owing to the intricate nature of intubating small animals, such as rats, most undergo procedures without mechanical ventilation, thus requiring the maintenance of their natural respiratory function (5). Consequently, there is a critical need for continuous monitoring of respiratory rate variability to ensure meticulous observation of anesthetic depth. This study aims to contribute to the 3Rs philosophy by developing a sophisticated non-contact vital sign measurement technique to maximally reduce animal suffering. A 24GHz microwave radar was applied to measure minute body surface displacements in the order of millimeters derived from respiration in laboratory animals to realize a novel non-contact vital sign monitoring system.

Several methods for monitoring respiratory and cardiac activities in rodents have been reported. An implantable radiotelemetry device was designed for mice that enabled accurate recordings of systolic, diastolic, and mean blood pressures, in addition to heart rate and locomotor activity (6). However, the procedure for the initial surgical implantation can result in stress, pain, and potentially chronic limitations in mobility, thereby posing a risk to animal welfare. Electrocardiography (ECG) and photoplethysmography (PPG) are non-invasive devices commonly used to monitor the vital signs of animals in experimental settings and provide valuable data on the status and function of the respiratory and cardiovascular systems (7, 8). ECG and PPG, though primarily for cardiovascular monitoring, also reflect respiratory activity. ECG captures respiratory-induced baseline variations from chest movements, while PPG exhibits modulations in amplitude and baseline due to respiratory effects on venous return, thoracic pressure, and arterial pulses, enabling respiratory waveform estimation via signal processing (9, 10). Although ECG and PPG provide detailed and direct measurements of respiratory and cardiovascular functions, their requirement for physical contact and the potential to stress or restrain the animals can limit their applicability in certain experimental settings.

In recent years, the push for the unobtrusive and contactless monitoring of vital signs in animal experiments has intensified to refine research methods and enhance animal welfare. Advances in noncontact vital sign measurement technologies have introduced innovative methods, such as thermal imaging (11, 12), remote photoplethysmography (rPPG) (7), radio frequency (RF) sensors (13–15), and piezoelectric sensors (16). Because body temperature can reflect several underlying physiological states, thermal imaging can be used to assess a range of vital signs, including body temperature, respiration rate, and potentially even heart rate (17). rPPG allows the measurement of heart rate and other cardiac-related information by analyzing the color changes in the skin that occur with the blood pulse. Piezoelectric sensors convert mechanical stresses (such as vibrations from heartbeats or breathing) into electrical signals without direct contact with the skin. These technologies offer significant benefits for monitoring vital signs without direct contact, enhancing comfort, and reducing stress in both humans and animals in clinical and research settings. Each of these technologies has unique advantages and potential applications in health-monitoring systems.

The objective of this study was to design and validate a noncontact monitoring system for assessing respiratory rate variability in anesthetized rats. The system can overcome numerous challenges encountered by traditional implantable sensors and contact methods used to monitor vital signs in rats. The study meets the demand for stress-free monitoring methods that align with the 3Rs principle in laboratory animal research. This study aims to achieve a detection accuracy of less than one bpm difference in respiratory rate using the non-contact monitoring system, compared to a reference laser device.

## 2 Methods

A radar module operating within the frequency range of 24.05 to 24.25 GHz was utilized to measure cardiopulmonary activity in rats. During respiration cycles, the expansion and contraction of the chest result in slight changes in the distance between the radar module and the rat’s body, causing phase or frequency shifts in the reflected microwaves. By analyzing these shifts, cardiopulmonary signals were extracted. To enable practical application, a standalone monitoring system centered on the radar module was developed using open-source hardware, including a single-board computer. The system’s software, designed on a Linux platform, facilitates data acquisition, signal processing, and visualization. To evaluate the system’s efficacy, experiments were conducted on four spontaneously breathing anesthetized rats. The following subsections detail the methodology:

### 2.1 Principle of microwave radar for measuring respiration signals

**Figure 1:**
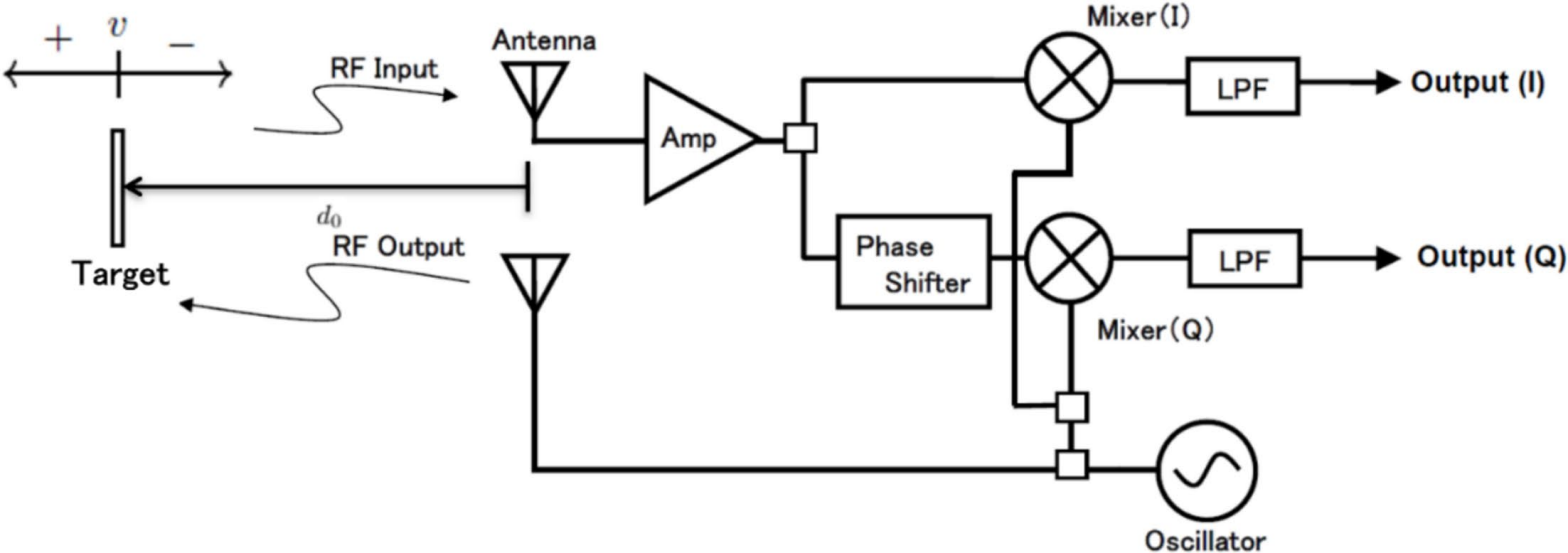
Principle of microwave radar for measuring respiration signals and block diagram of radar module.

As shown in Figure 1, the radar module consists of a transmitter and a receiver. The transmitter has a simple configuration that continuously transmits a specified frequency. In the receiver, the received wave is multiplied by two sine waves of the same frequency as the transmitted wave but with a phase difference of 90 degrees. This process separates the received wave into an in-phase (I) output and a quadrature (Q) output. The principle of employing microwave radar for noncontact measurement of respiration signals has been detailed in numerous publications (14, 18, 19). This section presents a brief overview of the simplified model. As shown in Figure 1, radio waves of frequency $f_{c}$ are transmitted to the target (i.e., the body surface of the rat). The transmitted signal is expressed as follows:


(1) $$s\left(t\right)=2\sin\left(2\pi f_{c}t\right)$$

Assume a case in which a target is moving at a speed of v (positive when receding, negative when approaching the radar) from a distance $d_{0}$ . The transmitted signal is reflected by the target, and the returned signal is registered as the received signal. With the speed of light set to $c=3\times 10^{8}m/s$ , the amplitude of the received signal is represented by $A\left(t\right)$ and can be described as follows.


(2) $$\begin{array}{c} r\left(t\right)=A\left(t\right)\sin\left(2\pi f_{c}\left(t-\frac{2\left[d_{0}+vt\right]}{c}\right)\right)\\ =A\left(t\right)\sin\left(2\pi \left(f_{c}-\frac{2f_{c}v}{c}\right)t-\frac{4\pi f_{c}d_{0}}{c}\right) \end{array}$$

In Figure 1 and Equation 2, the movement of the target is constant; however, it is generalized based on the assumption of respiratory signals. Such biological movements associated with respiration can be considered as the target moving back and forth periodically at a constant distance $d_{0}$ from the radar. Therefore, in Equation 2, vt can be replaced with $d\left(t\right)$ .


(3) $$vt\to \overset{t}{\underset{0}{\int }}v\left(t\right)dt\to d\left(t\right)$$

The signal $r\left(t\right)$ is multiplied by $2\cos\left(2\pi f_{c}t\right)$ or $2\sin\left(2\pi f_{c}t\right)$ respectively, and the signals that pass through the low-pass filter become the detected signals $I\left(t\right)$ and $Q\left(t\right)$ .


(4) $$\begin{array}{c} I\left(t\right)=LPF\left[2\cos\left(2\pi f_{c}t\right)r\left(t\right)\right]\\ =LPF\left[\begin{array}{l} A\left(t\right)\sin\left(4\pi f_{c}t-2\pi \frac{2f_{c}d\left(t\right)}{c}-\frac{4\pi f_{c}d_{0}}{c}\right)\\ -A\left(t\right)\sin\left(2\pi \frac{2f_{c}d\left(t\right)}{c}+\frac{4\pi f_{c}d_{0}}{c}\right) \end{array}\right]\\ \begin{array}{l} =-A\left(t\right)\sin\left(\frac{4\pi f_{c}d\left(t\right)}{c}+\frac{4\pi f_{c}d_{0}}{c}\right)\\ =A\left(t\right)\cos\left(\frac{4\pi f_{c}d\left(t\right)}{c}+\frac{4\pi f_{c}d_{0}}{c}+\frac{\pi }{2}\right) \end{array} \end{array}$$

$$\begin{array}{c} Q\left(t\right)=LPF\left[2\sin\left(2\pi f_{c}t\right)r\left(t\right)\right]\\ =LPF\left[\begin{array}{l} A\left(t\right)\cos\left(2\pi \frac{2f_{c}d\left(t\right)}{c}+\frac{4\pi f_{c}d_{0}}{c}\right)\\ -A\left(t\right)\cos\left(4\pi f_{c}t-2\pi \frac{2f_{c}d\left(t\right)}{c}-\frac{4\pi f_{c}d_{0}}{c}\right) \end{array}\right]\\ \begin{array}{l} =A\left(t\right)\cos\left(\frac{4\pi f_{c}d\left(t\right)}{c}+\frac{4\pi f_{c}d_{0}}{c}\right)\\ =A\left(t\right)\sin\left(\frac{4\pi f_{c}d\left(t\right)}{c}+\frac{4\pi f_{c}d_{0}}{c}+\frac{\pi }{2}\right) \end{array} \end{array}$$

In Equation (4), $I\left(t\right)$ and $Q\left(t\right)$ are assumed to include phase shift due to the propagation and passage through the antenna, wires, and circuits after reception, appearing as the residual phase ∅ . Additionally, the frequency $f_{c}$ is converted to the wavelength $\lambda_{c}$ . Consequently, $I\left(t\right)$ and $Q\left(t\right)$ can be expressed as follows.


(5) It=Atsin2πdtλc2+2πd0λc2+∅Qt=Atcos2πdtλc2+2πd0λc2+∅

### 2.2 Configuration of non-contact vital signs monitoring system

**Figure 2:**
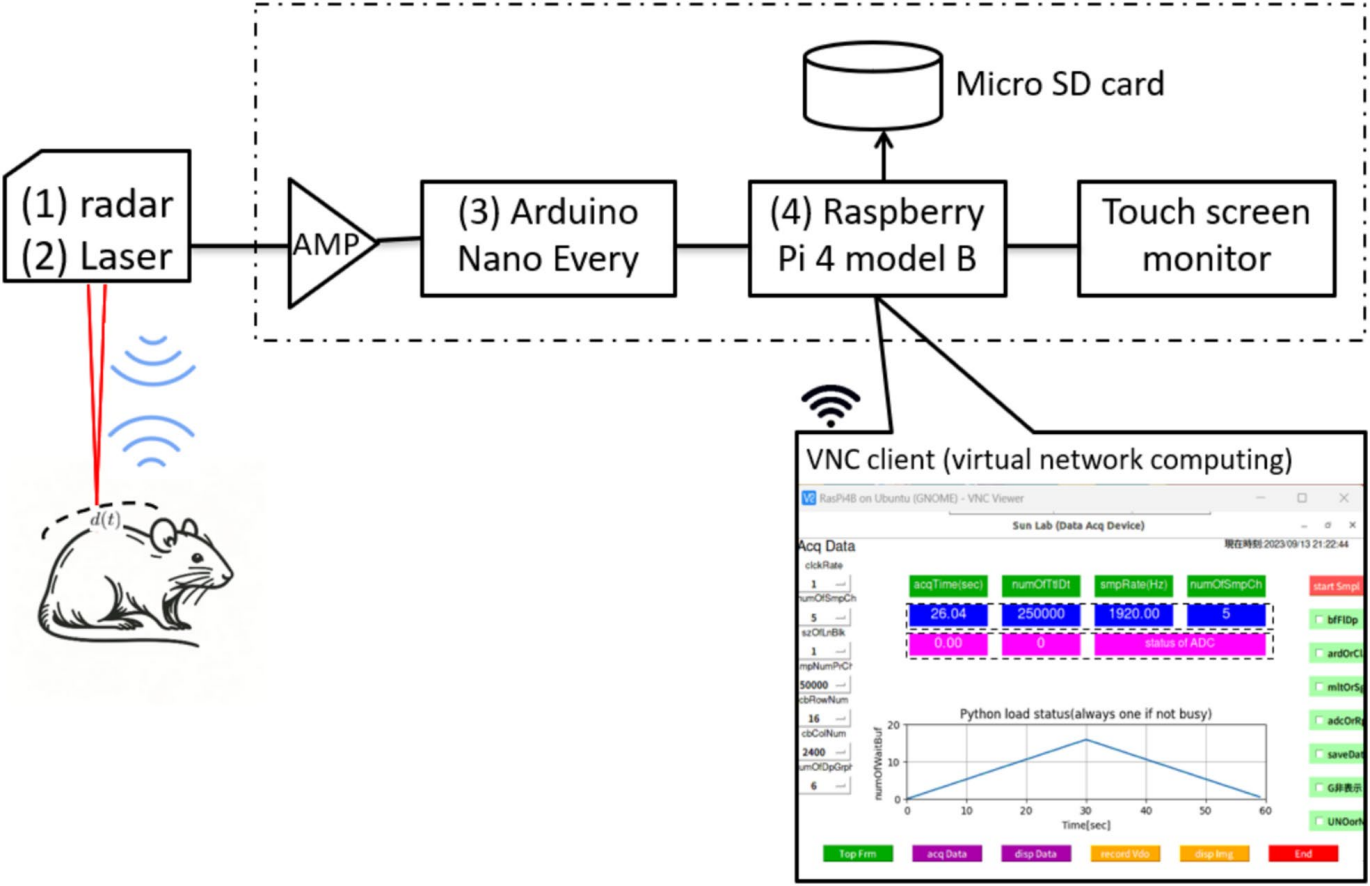
Configuration of non-contact vital signs monitoring system.

The configuration of the noncontact vital sign monitoring system is shown in Figure 2 and consists of four parts. (1) We utilized a 24.05 to 24.25 GHz I/Q channel microwave radar equipped with an 8-element TX/RX antenna (SHARP, model DC6M4JN3000, Japan). Notably, the open frequency band does not require user licensing, making it uniquely permissible for microwave sensors under specified low-power conditions in both indoor and outdoor applications. (2) We concurrently utilized a CMOS multi-function analog laser sensor (Keyence, models IL-1000 & IL-S065, Japan) with a repeatability accuracy of 2 μm to measure chest wall displacement during respiration. (3) An Arduino data acquisition board was used to convert the I/Q and laser signals into digital signals with a sampling rate of 1,092 Hz and a resolution of 10 bits. (4) The entire system is controlled by a single-board computer that utilizes a 64-bit quad-core Cortex-A72 processor and 8 GB RAM, specifically the Raspberry Pi 4 model B, developed by the Raspberry Pi Foundation in the United Kingdom.

The software, developed entirely on an open-source Python platform, comprises four main modules: a data acquisition module implemented with Arduino IDE C programming, a data analysis module for calculating respiratory rate variability, a waveform display module for presenting respiration curves, and a wireless communication module that facilitates client device connectivity (including laptops, tablets, and smartphones) via virtual network computing technology.

### 2.3 Animal and protocol

**Figure 3:**
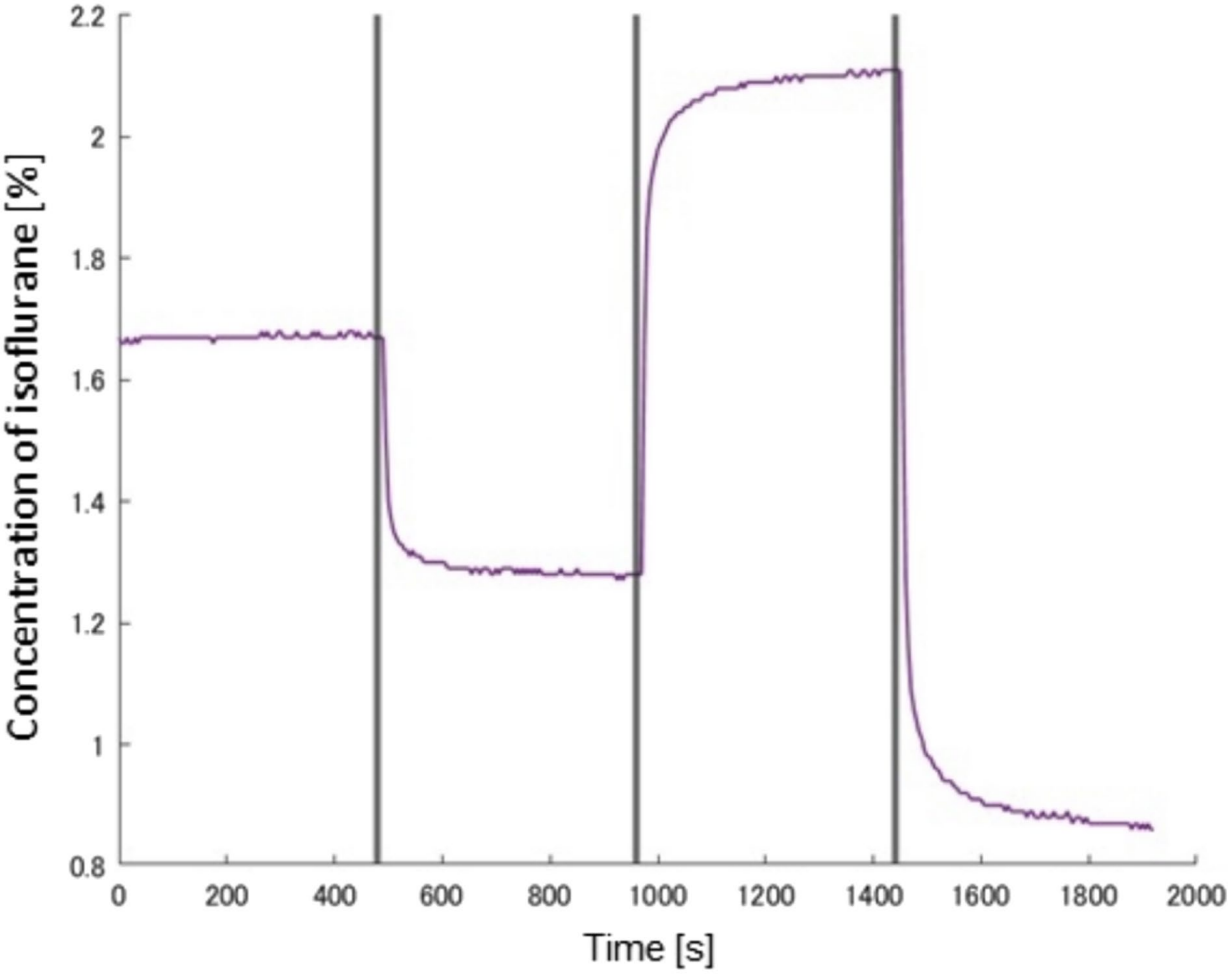
A time profile of isoflurane concentration changes during a preliminary experiment.

Male Wistar rats (*n* = 4, 10–19 weeks of age, body mass 216–334 g, Japan SLC, Shizuoka, Japan) were used in this study. The rats were maintained on a 12:12-h light–dark cycle and received food and water ad libitum. All experiments were approved by Animal Ethics Committee of the University of Electro-Communications (Approval number: A44) and were conducted in full compliance with the ARRIVE guidelines. The same trained technician performed the anesthesia, skin disinfection, skin incision, carotid artery dissection, and catheter insertion in the rats. Rats were anesthetized with 2–3% isoflurane (Pfizer Japan, Tokyo, Japan) at airflow rate of 0.8 L/min via a face mask using anesthetic vaporizer with a built-in ventilation device (NARCOBIT-E, Natsume Manufacturing, Tokyo, Japan). After disinfection of the skin surface with 70% ethanol, an incision was made on the ventral side of the neck, and the right carotid artery was isolated. A PE-50 catheter (Polyethylene Tubing; Natsume Seisakusho Tokyo, Japan) filled with heparinized saline was inserted in the right carotid artery to record mean arterial blood pressure (MAP) via a pressure transducer (DX-100; Nihon Kohden, Tokyo, Japan). The pressure signals were continuously sampled at 1 kHz with a PowerLab and recorded on a computer using LabChart software (AD Instruments, Colorado Springs, CO). The average blood pressure at the start of measurement was 97.3 ± 12.0 mm Hg, which confirms the physiological range of normal rats. A preliminary experiment was conducted, at baseline, air with 1.7% isoflurane was inhaled using the anesthetic vaporizer (Natsume, Tokyo, Japan), and the concentration was changed to 1.3, 2.1 and 0.9% at 8-min intervals (Figure 3). The INHALATION AGENT concentration was measured by a monitoring system (FI-8000, Riken Keiki, Tokyo, Japan). In this experiment, we maintained a gas flow rate of 0.8 L/min and varied only the anesthetic concentration. We measured the gas concentration inside the mask, taking into account the dead space that occurs in the tube between the anesthetic device and the rat’s mask. As a practical measurement, the time to change isoflurane gas concentration was less than 60 s (Figure 3). Body temperature was maintained near 37°C by placing the animal on a heating pad during surgery. At the end of the experimental protocol, animals were euthanized using an isoflurane overdose. We have shared the dataset on an open-access platform (20). It can be accessed at https://data.mendeley.com/datasets/swk27btvgd/1.

### 2.4 Signal processing to analysis of instantaneous respiration frequency

**Figure 4:**
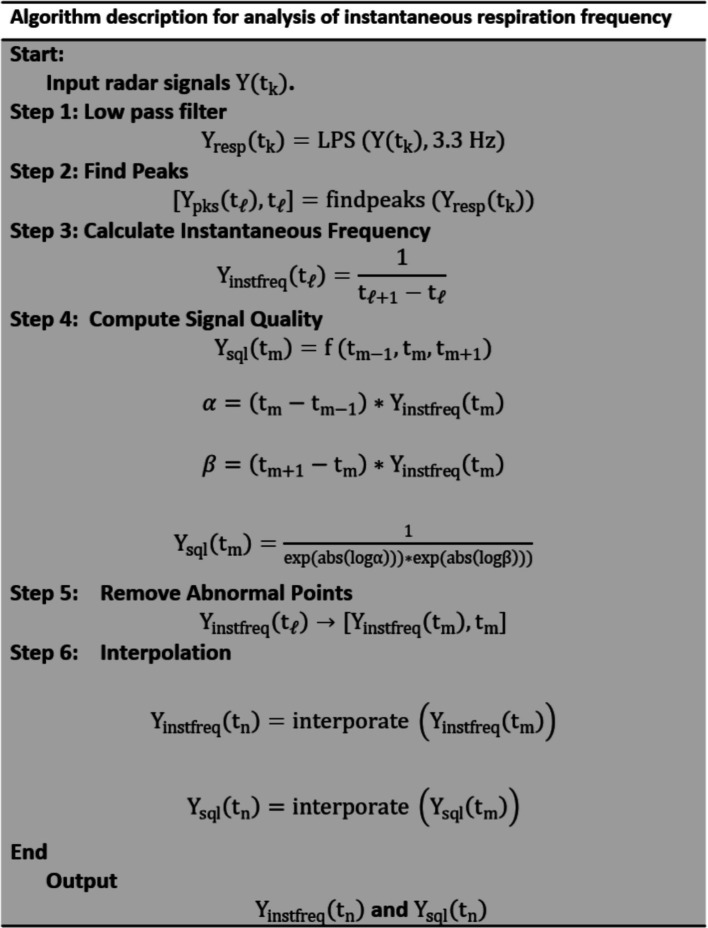
Algorithm chart for analysis of instantaneous respiration frequency.

A flowchart of the signal processing for analyzing radar signals to calculate instantaneous respiration frequency is shown in Figure 4. $Y\left(t_{k}\right)$ is the input radar signal or reference laser signal at time $t_{k}$ . It can take the form $I\left(t_{k}\right)$ , $Q\left(t_{k}\right)$ , or $R\left(t_{k}\right)\text{.}$ A low-pass filter (LPF) with a cutoff frequency of 3.3 Hz is applied to the radar signals $I\left(t_{k}\right)$ and $Q\left(t_{k}\right)$ to obtain the respiration signal $Y_{\mathrm{resp}}\left(t_{k}\right)$ . However, the reference signal, $R\left(t_{k}\right)$ does not pass through the LPF.


(6) $$Y_{\mathrm{resp}}\left(t_{k}\right)=LPS\;\left(Y\left(t_{k}\right),3.3\;\mathrm{Hz}\right)$$

The peaks in $Y_{\mathrm{resp}}\left(t_{k}\right)$ are detected to determine [ $Y_{pks}(t_{l}$ ), $t_{l}$ ], where $t_{l}$ are the times at which the peaks occur. The instantaneous respiration frequency, $Y_{\mathrm{instfreq}}\left(t_{l}\right)$ is calculated as the reciprocal of the time difference between consecutive peaks.


(7) $$\left[Y_{pks}\left(t_{l}\right),t_{l}\right]=\mathrm{findpeaks}\;\left(Y_{\mathrm{resp}}\left(t_{k}\right)\right)$$

(8) $$Y_{\mathrm{instfreq}}\left(t_{l}\right)=\frac{1}{t_{l+1}-t_{l}}$$

The abnormal points $Y_{\mathrm{instfreq}}\left(t_{l}\right)$ are removed based on the signal quality assessment from $Y_{\mathrm{instfreq}}\left(t_{l}\right)$ to obtain a clean-up time series. The signal quality is assessed at each calculated frequency point. The differences in the interval times between consecutive measurements are used to compute a quality metric, $Y_{sql}\left(t_{m}\right)$ , which reflects the consistency of the frequency measurement.


(9) $$Y_{sql}\left(t_{m}\right)=f\;\left(t_{m-1},t_{m},t_{m+1}\right)$$

(10) $$\alpha =\left(t_{m}-t_{m-1}\right)*Y_{\mathrm{instfreq}}\left(t_{m}\right)$$

(11) $$\beta =\left(t_{m+1}-t_{m}\right)*Y_{\mathrm{instfreq}}\left(t_{m}\right)$$

(12) $$Y_{sql}\left(t_{m}\right)=\frac{1}{\exp\left(\mathrm{abs}\left(\mathrm{log\alpha}\right)\right)*\exp\left(\mathrm{abs}\left(\mathrm{log\beta}\right)\right)}$$

(13) $$Y_{\mathrm{instfreq}}\left(t_{l}\right)\to \left[Y_{\mathrm{instfreq}}\left(t_{m}\right),t_{m}\right]$$

Interpolation is applied to generate a uniformly spaced time series for both $Y_{\mathrm{instfreq}}\left(t_{m}\right)$ and $Y_{sql}\left(t_{m}\right)$ . This step ensures that the data points are evenly distributed for further analysis or visualization.


(14) $$Y_{\mathrm{instfreq}}\left(t_{n}\right)=\mathrm{interporate}\;\left(Y_{\mathrm{instfreq}}\left(t_{m}\right)\right)$$

(15) $$Y_{sql}\left(t_{n}\right)=\mathrm{interporate}\;\left(Y_{sql}\left(t_{m}\right)\right)$$

(16) $$Y_{\mathrm{instbpm}}\left(t_{n}\right)=Y_{\mathrm{instfreq}}\left(t_{n}\right)*60$$

### 2.5 Statistical analysis

Bland–Altman and Pearson’s correlation analyses were employed to compare the radar with the reference laser measurements. The Bland–Altman analysis, recognized for its simplicity and efficiency, was utilized to evaluate the agreement between the two measurements in clinical studies. The measurement accuracy was assessed using the root mean square error (RMSE) and mean absolute percentage error (MAPE). A power analysis was conducted to determine the minimum sample size required with 80% power and a 5% significance level. Additionally, the Kruskal–Wallis and post-hoc tests were used to analyze the differences in respiratory rate variability across the four distinct isoflurane levels. Statistical significance was set at *p* < 0.05.

## 3 Results

### 3.1 Comparative assessment of respiration rate obtained via radar and laser

**Figure 5:**
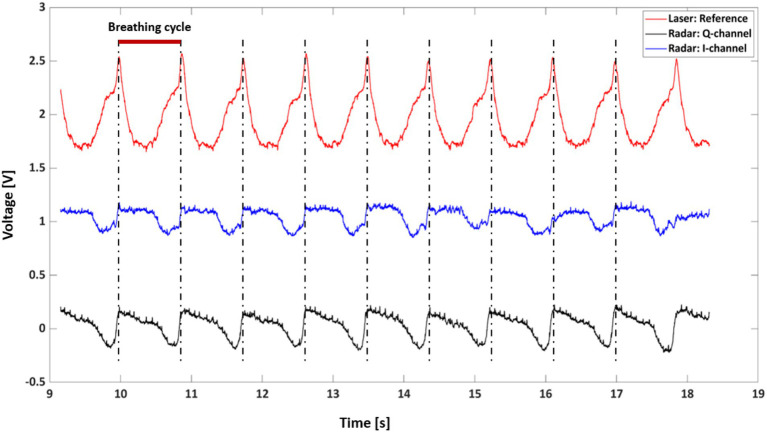
Example of respiratory signals obtained via radar and laser.

Figure 5 provides a comparative analysis of respiratory signals captured by the radar and laser measurement systems over a 10-s interval from rat A. Three distinct waveforms are depicted, with the laser-based signal serving as a reference (red line) and the radar-derived signals represented by the Q-channel (blue line) and I-channel (black line). These signals are characterized by periodic peaks and troughs that align with the physiological phenomena of inhalation and exhalation.

The alignment of the radar signal peaks with the laser reference signal peaks indicates a strong correlation between the two measurement modalities, suggesting that the radar system reliably captures the rhythmic respiratory patterns. The red bar indicates a breathing cycle duration of approximately 900 ms, which is equivalent to a respiratory rate of 54 breaths/min (bpm).

**Figure 6:**
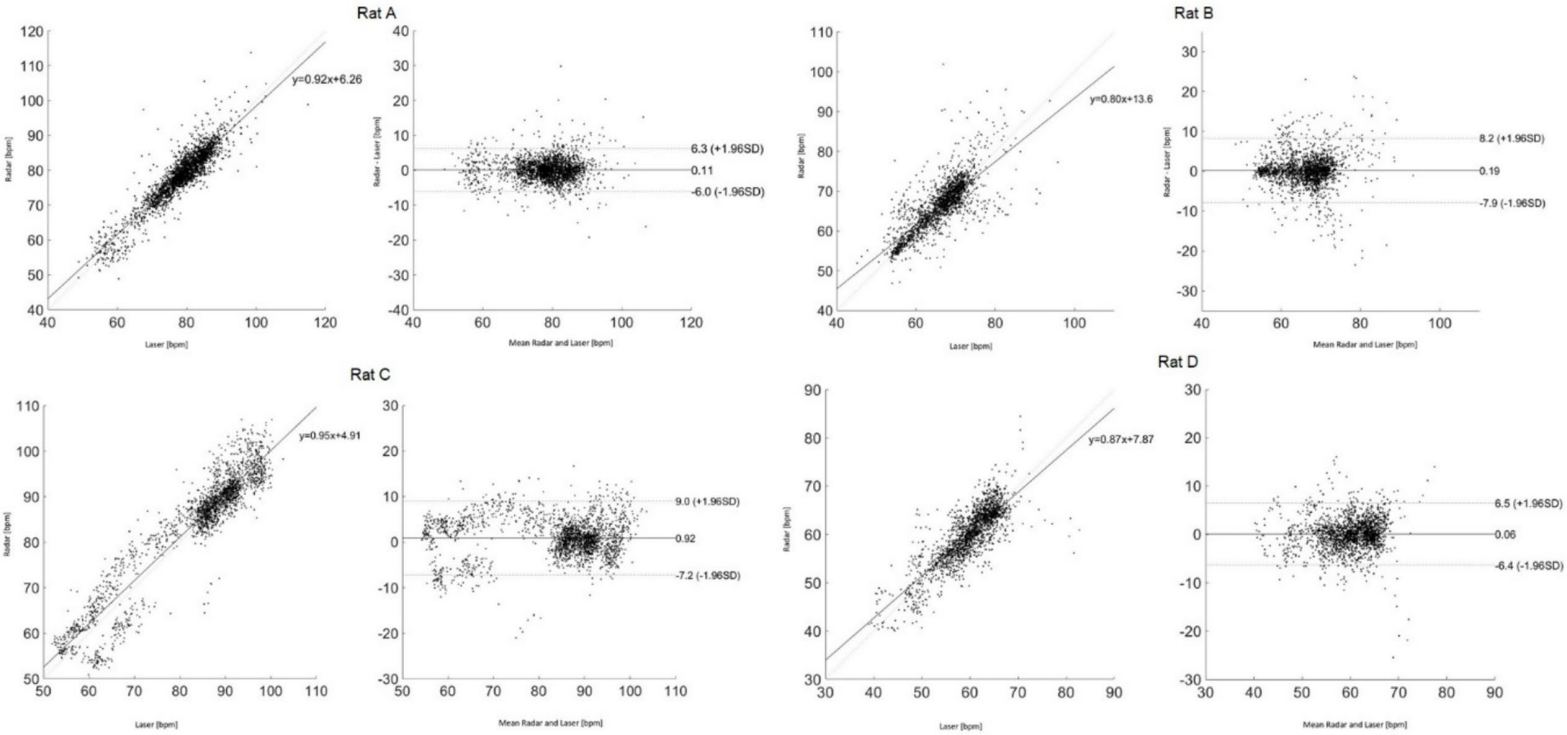
Bland–Altman and Pearson’s correlation plots based on measurements from rat **(A–D)**.

Figure 6 presents the Bland–Altman and Pearson’s correlation plots based on measurements from rat A to D. The measurement of Rat A comprises 2,525 pairs of respiratory rates, monitored under varying depths of anesthesia ranging from 2.5 to 1.0% across a 32-min period, reflecting changes in four different isoflurane levels. The analysis revealed a mean discrepancy in respiratory rate between the reference and radar of 0.11 bpm, with a 95% agreement limit spanning from −6.0 to 6.3 bpm. Pearson’s correlation coefficient for this set was 0.92 (*p* < 0.05), indicating a strong linear association. Additionally, the accuracy of the measurements was rigorously evaluated using the RMSE and MAPE to ascertain the reliability of the respiratory rate assessments in rats. The RMSE, a metric that quantifies the average magnitude of the error between the radar and laser values, was recorded at 3.1 bpm, indicating minimal deviation and, thus, a high degree of accuracy in the measurements. The MAPE, which expresses the error as a percentage of the radar value, was determined to be 2.8%. This relatively low percentage indicated that the deviation between the measured and actual respiratory rates was within a 5% margin. Table 1 summarizes the statistical results for the four rats.

**Table 1:** Statistical analysis and comparison of results for each rat.

	Rat A	Rat B	Rat C	Rat D
Root mean square error (RMSE)	3.1	4.1	8.5	3.2
Mean absolute percentage error (MAPE)	2.8%	3.8%	8.1%	3.9%
Correlation coefficient	0.92	0.80	0.95	0.87
The 95% limit of agreement range [bpm]	−6.0 to 6.3	−7.9 to 8.2	−7.2 to 9.0	−6.4 to 6.5
The mean difference [bpm]	0.1	0.19	0.92	0.06

### 3.2 Changes in respiratory rate variability on isoflurane anesthesia depth

**Figure 7:**
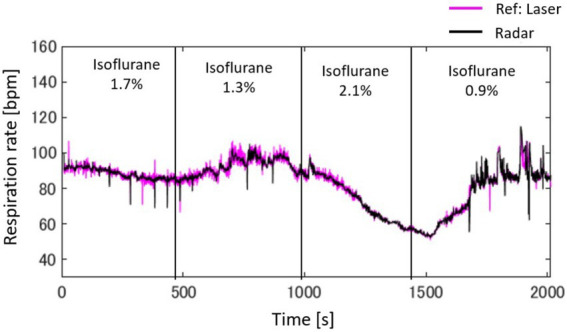
Radar-based assessment of respiratory rate fluctuations, highlighting how changes in isoflurane concentrations, beginning with 1.7%, progressing to 1.3%, then to 2.1%, and concluding at 0.9%, influence breathing patterns over a span of 32 min.

Figure 7 presents a radar-based assessment of respiratory rate fluctuations, highlighting how changes in isoflurane concentrations in rat A, beginning at 1.7%, progressing to 1.3%, then to 2.1%, and concluding at 0.9%, influenced the breathing patterns over a span of 32 min. Starting with 1.7% isoflurane, the respiration rate initially appeared to be stable at approximately 90 bpm but then exhibited a slight decrease. When the concentration was reduced to 1.3%, the rate stabilized. Increasing isoflurane to 2.1% corresponded to a sharp decrease in respiration rate, dropping to just above 50 bpm. Finally, lowering the concentration to 0.9% was associated with a rapid increase in the respiration rate, returning to approximately 80 bpm. The figure compares the laser and radar methodologies for measuring the respiration rate, represented by pink and black lines, respectively. A close alignment between the radar data and the laser reference indicates that radar can be observed.

**Figure 8:**
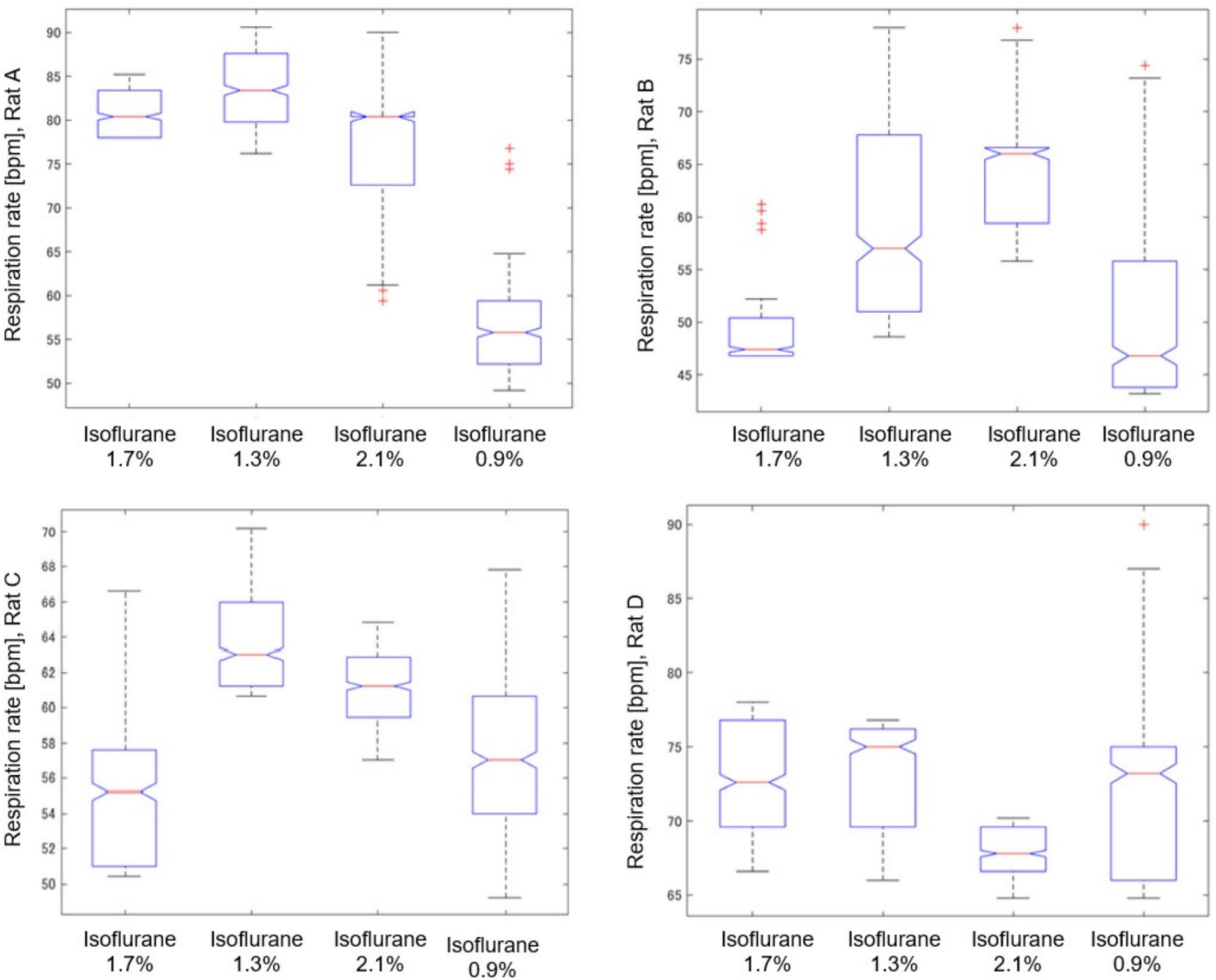
Impact of isoflurane concentrations on respiration rate in rat **(A–D)** through a box plot analysis over a 32-min period.

Figure 8 provides a detailed analysis of the impact of varying isoflurane concentrations on the respiration rate of rat A to D over a 32-min period, presented as a box plot. These data underscore the sensitivity of respiratory function to changes in isoflurane levels, confirming its potent depressive effect at elevated concentrations.

As shown in Table 2, we analyzed the effects of different isoflurane concentrations on respiratory rate fluctuations in four rats (A, B, C, and D) using radar-based assessments over a 32-min period. The results, derived from Kruskal–Wallis tests followed by post-hoc comparisons, revealed statistically significant differences across various concentrations for all rats, except for Rat A at a concentration change from 2.1 to 1.3%, which was not significant. However, further increases to 2.1% and reductions to 0.9% from the initial 1.7% concentration showed highly significant effects on the respiratory rates of all subjects.

**Table 2:** Effects of isoflurane concentration changes on respiratory rate fluctuations in rats: Kruskal–Wallis and *post-hoc* analysis results.

	Kruskal–Wallis	*Post-hoc* comparisons		
		Iso-1.7 vs. iso-1.3	Iso-1.7 vs. iso-2.1	Iso-1.7 vs. iso-0.9
Rat A	***	ns	***	***
Rat B	***	***	***	***
Rat C	***	***	***	***
Rat D	***	***	***	***

*p < 0.05; **p < 0.01; ***p < 0.001; ns, not significant.

## 4 Discussion

In this study, we proposed and explored the development of a noncontact vital sign monitoring system designed to enhance the welfare of laboratory animals. Traditional methods predominantly rely on direct visual inspection or the use of restraining devices during the measurement process (21, 22) and the interrogation of implanted tags (23, 24). The limited adoption of non-contact monitoring technologies in laboratory settings can be attributed to several physiological and behavioral characteristics of these animals. These include their relatively small size, fur coverage, constant movement, and significantly higher vital signs compared to those of humans, which complicate the development and implementation of non-contact monitoring systems. Our study addresses these barriers by proposing a novel, noninvasive approach for measuring vital signs without physical contact.

The technical contributions of this study can be summarized as follows. (1) The system was engineered using open-source hardware components, including Raspberry Pi and Arduino, which were integrated using software running on a Linux platform. This integration facilitates comprehensive functions, such as data acquisition, signal processing, and real-time visualization. The use of open-source platforms not only ensures cost-efficiency and modifiability but also enhances the accessibility of the technology for broad-based research applications. (2) Advanced signal processing techniques were implemented to analyze the phase and frequency changes in the microwaves reflected from the rat’s body owing to cardiopulmonary activity. This enabled the accurate calculation of instantaneous respiration frequencies. The process involved filtering, peak detection, and quality assessment of the radar signals to ensure precise and reliable measurement of the respiratory rates. (3) This study provides insights into how varying depths of anesthesia, controlled by the administration of isoflurane, affect the respiratory rate variability in rats. The direct effect of anesthesia depth on respiratory dynamics was highlighted by methodically reducing the isoflurane concentration from 2.5 to 1.0%. For example, The Rat A starting at a concentration of 1.7%, the respiration rate hovered at approximately 90 bpm and decreased slightly upon reduction to 1.3%, indicating stability. A further increase to 2.1% resulted in a significant decline in the respiratory rate to just above 50 bpm, indicating a pronounced depressive effect at higher concentrations. Conversely, reducing isoflurane to 0.9% led to an increase in the respiration rate to approximately 80 bpm, suggesting a return toward baseline as the influence of the anesthetic diminished. These findings highlight the critical influence of changes in isoflurane concentration on respiratory dynamics, with potential implications for anesthesia management in clinical and research settings.

This study primarily aimed to demonstrate the feasibility of measuring respiratory rate variability in rats using radar technology, with a focus on developing a non-contact vital sign measurement system. For the evaluation of measurement accuracy, we used a laser sensor as the reference device (15). The choice of the laser sensor was guided by several considerations: (1). Unlike contact-based methods such as ECG, respiratory bands, or airflow sensors, laser sensors impose minimal physical burden on the subjects, which is particularly important in experiments involving rats under anesthesia. (2). Laser sensor allow for evaluation and experimentation in alignment with the measurement principles of radar, making it as a suitable comparison tool. Recall the Equation 5, the chest wall displacement caused by respiration, $d\left(t\right)$ , can be directly measured using a laser sensor. The radar signal outputs, I(t) and Q(t), maintain linearity with respect to $d\left(t\right)$ . This alignment between radar and laser measurement principles underscores the suitability of the laser sensor for validating radar-based respiratory measurements. For the purpose of evaluating measurement accuracy against the reference laser device, we considered data collected from four rats under four different anesthesia conditions to be sufficient. Additional evaluations involving a larger number of rats will be necessary in future studies to investigate the physiological aspects of this system.

## 5 Conclusion

The implementation of such a system can radically transform the monitoring process by making it simpler, more precise, and less invasive. This would not only improve the daily management of laboratory animals by providing stress-free, quantitative assessments of their health but also enhance overall animal welfare. Furthermore, in pharmacological and pharmacodynamic research, the ability to measure vital signs without inducing stress can minimize data variability. Thus, the implications of this technology extend beyond animal welfare and influence the broader field of biomedical research.

## Data Availability

The datasets presented in this study can be found in online repositories. The names of the repository/repositories and accession number(s) can be found in the article/supplementary material.
